# Supplementary material for: Splice-Junction-Based Mapping of Alternative Isoforms in the Human Proteome
Source: Cell Rep. Author manuscript; Available in PMC 2020 Jan 15. (PMC6961840; doi:10.1016/j.celrep.2019.11.026)
Supplement: 2 [file NIHMS1546469-supplement-2.zip › DF1/PXD010154/Testis/Testis_7_FDXR_TPQICVVGSGPAGFYTAQHLLKG.pdf]

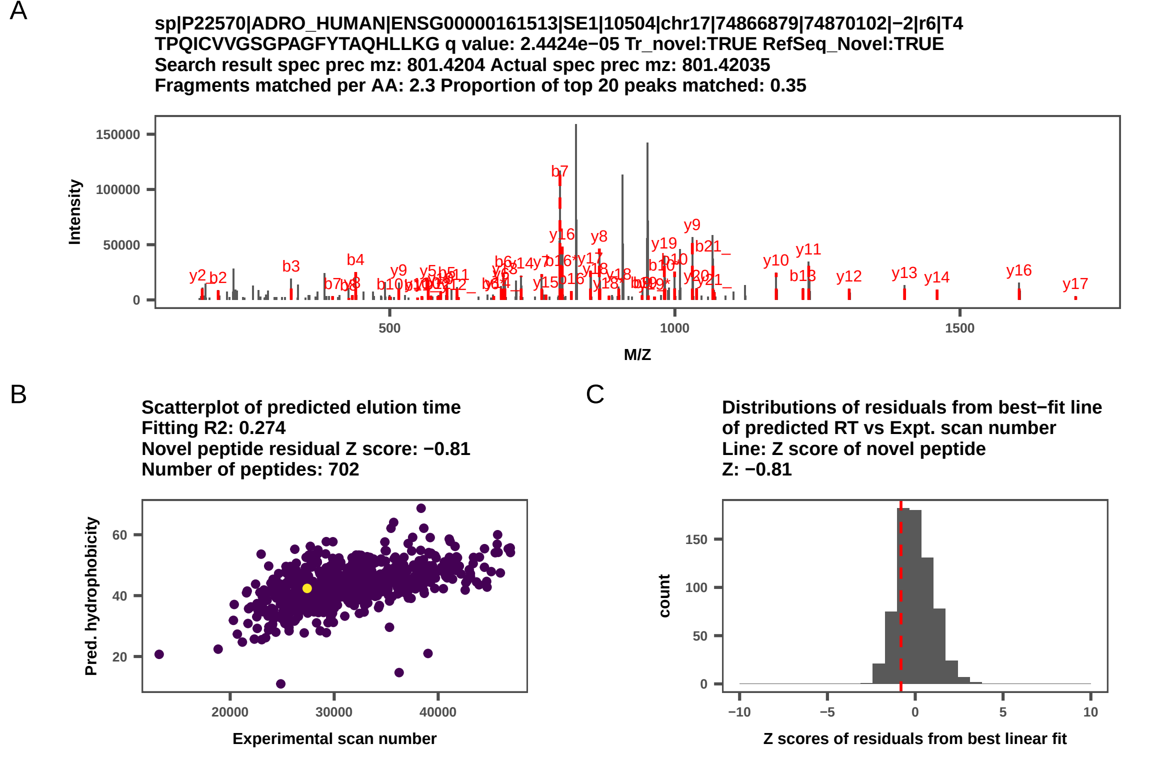
A
sp|P22570|ADRO_HUMAN|ENSG00000161513|SE1|10504|chr17|74866879|74870102|−2|r6|T4
TPQICVVGSGPAGFYTAQHLLKG q value: 2.4424e−05 Tr_novel:TRUE RefSeq_Novel:TRUE
Search result spec prec mz: 801.4204 Actual spec prec mz: 801.42035
Fragments matched per AA: 2.3 Proportion of top 20 peaks matched: 0.35
Intensity
150000
100000
50000
0
500
1000
1500
M/Z
B
Scatterplot of predicted elution time
Fitting R2: 0.274
Novel peptide residual Z score: −0.81
Number of peptides: 702
Pred. hydrophobicity
60
40
20
20000
30000
40000
Experimental scan number
C
Distributions of residuals from best−fit line
of predicted RT vs Expt. scan number
Line: Z score of novel peptide
Z: −0.81
count
150
100
50
0
−10
−5
0
5
10
Z scores of residuals from best linear fit
